# In vitro evaluation of different protective techniques to reduce titanium particle contamination during implantoplasty

**DOI:** 10.1007/s00784-023-05037-8

**Published:** 2023-05-04

**Authors:** A. Platt, C. C. Liu, A. Gubler, N. Naenni, D. Thoma, P. R. Schmidlin

**Affiliations:** 1 Clinic of Conservative and Preventive Dentistry, Division of Periodontology and Peri-Implant Diseases, Center of Dental Medicine, University of Zurich, 8032 Zurich, Switzerland; 2 Clinic of Reconstructive Dentistry, Center of Dental Medicine, University of Zurich, 8032 Zurich, Switzerland

**Keywords:** Implantoplasty, Particle contamination, Titanium

## Abstract

**Objectives:**

Our aim is to study titanium remains in a bone model during standardized implantoplasty under different isolation and protective modalities.

**Material and methods:**

Forty implants were placed in artificial spongy bone blocks mimicking a horizontal bone loss and implant neck protrusion of 5 mm. Samples were randomly divided into four groups (*n* = 10), which were treated as follows: rubber dam (A), a dental adhesive paste (B), bone wax (C), and an unprotected positive control (D). Implantoplasty was performed using carbide and diamond burs under strict water cooling and standardized suction. After removal of the respective isolation materials, the bone blocks were thoroughly rinsed with tap water for 3 min and titanium chips were collected using a filter integrated in the model. The filter paper was removed and dissolved in 37% hydrochloric acid for 2 h at 120 °C and the titanium remnants were quantified using atomic absorption spectrometry.

**Results:**

None of the test groups were able to completely prevent titanium particle contamination. Rubber dam (691 ± 249 µg) and bone wax (516 ± 157 µg) were found to be significantly more protective than the positive control (2313 ± 747 µg) (*p* < 0.001) with respect to the amount of titanium particles that remained in the bone model after implantoplasty. The adhesive paste group (1863.5 ± 538 µg) was not significantly different from the positive control (*p* = 0.19).

**Conclusions:**

Despite some limitations of the present study, titanium particles resulting from a standardized implantoplasty can be assumed to be significantly reduced when the tissues/bone were protected with rubber dam and bone wax, or a combination, depending on individual accessibility.

**Clinical relevance:**

Tissue protective measures to reduce or avoid particle contamination during implantoplasty is possible and should be considered and further clinically assessed to avoid iatrogenic inflammatory reactions.

## Introduction

Dental implants have become an increasingly popular method for replacing teeth after tooth loss [1]. Although they have a high survival rate, biological complications can lead to peri-implant diseases such as peri-implant mucositis and peri-implantitis. Reversible inflammation in the soft tissues around an implant is known as peri-implant mucositis and if left untreated, the inflammatory process can lead to destructive and progressive loss of supporting bone in the development of peri-implantitis [2]. This inflammatory lesion is caused by plaque bacteria that accumulate on the implant threads and form a biofilm that is difficult for access and home hygiene. Prevalence of peri-implantitis reported in the literature varies from 4 to 45% [3, 4].

Peri-implantitis treatment with only non-surgical treatment has its limitations due to the complex implant architecture, difficult access, and defect anatomy. Therefore, more invasive treatments are usually required. Possible surgical treatments include resective or implantoplasty and/or regenerative approach [5]. There is some evidence in the literature to recommend implantoplasty as a possible treatment for peri-implantitis. A meta-analysis showed that implantoplasty contributed to significant improvement of the peri-implant condition in terms of reduced probing depth, bleeding on probing, and suppuration after treatment [6].

During this mechanical procedure, the exposed implant threads are removed and a smooth and polished surface is created to efficiently clean the implant surface and to prevent bacteria to adhere further [7]. However, significant debris such as titanium or material particles are produced in the process, which can only be partially rinsed off or sucked off. These titanium particles can be deposited especially in the porous bone but also in the soft tissue and themselves cause an inflammatory reaction. A recent study analyzed histopathological findings in human soft tissue biopsies of implants with peri-implantitis and showed the presence of foreign bodies surrounded by chronic inflammatory infiltrates [8]. The foreign bodies were predominantly titanium and dental cement. In another study, metal-like particles and associated macrophages were detected in swabs of peri-implant mucosa from clinical specimens. These were detected in both, i.e., patients with and without peri-implantitis, with a higher concentration of titanium in peri-implantitis [9]. Higher levels of titanium have also been found in the dental plaque of patients with peri-implantitis compared to patients without peri-implantitis [10]. In vitro studies demonstrated the influence of titanium particles on macrophages, which react with an excessive upregulation of the pro-inflammatory cytokines TNF-α, IL-1β, and IL-6, key molecules in bone remodeling [11–13], and have been shown to induce activation and secretion specifically of IL-1β from macrophages [14]. Further studies have elucidated that titanium dissolution products could also negatively influence the peri-implant microbiome structure and diversity [15]. While titanium particles can derive already from implant placement or corrosion processes [8, 16], iatrogenic measures as extensively performed during implantoplasty produce particles and are therefore a suggested meaningful source of a trigger for a greater immune response. To prevent such titanium being released into the tissues during therapeutic instrumentations, either implantoplasty should be abandoned in first place or methods are required to efficiently protect the surrounding tissues.

Taking all this into account, this study aimed to investigate three different types of physically protective barriers using a novel implant–bone model simulating the conditions during implantoplasty in vitro to reduce particle penetration in the bone. We hypothesized that particles can be prevented from entering cancellous bone during a standardized implantoplasty.

## Materials and methods

### In vitro* model preparation*

**Fig. 1:**
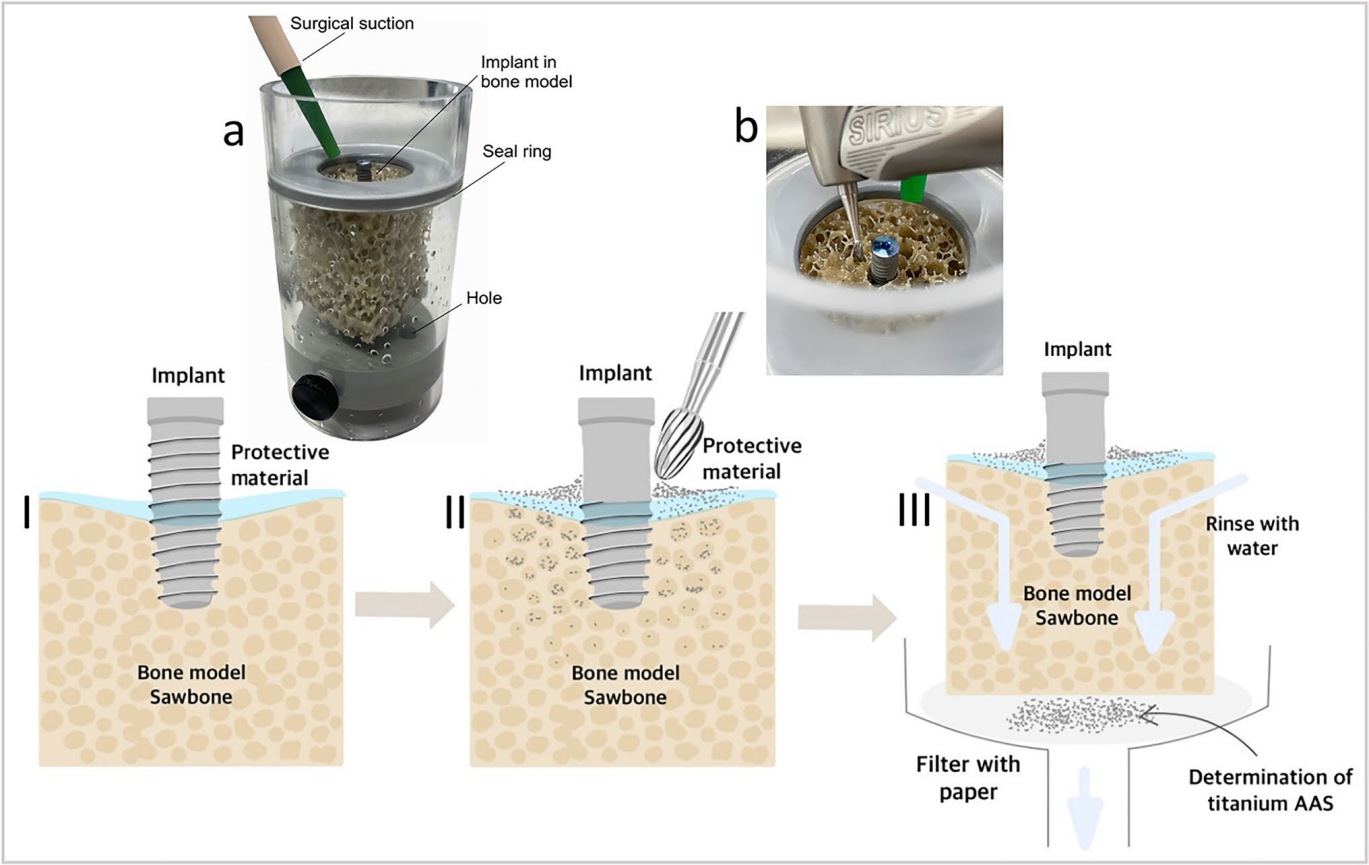
Illustration and overview of the experimental set-up and treatment steps (a/b). I. Application of the protective materials to the bone model. II. Performance of the implantoplasty. III. Collection of the titanium particles

This study was performed in a custom-made set-up with a rectangular bone block model held in a Plexiglas cylinder (Fig. 1a). First, preparation of these bone blocks involved pre-drilling 6.5-mm deep with a drill (3.9 mm diameter, Emco FB-2 drilling and milling machine; Maier & Co, Hallein, Austria) so that the titanium implants (T3 Non-Platform Switched Tapered Implant 4 × 11.5 mm, REF BOST411; Biomet 3i, Spain) protruded 5 mm. To simulate osseointegration, the implants were fixed in the bone model with a small drop of superglue (UHU superglue; UHU GmbH & Co. KG, Bühl Germany).

The Plexiglas cylinder model was constructed in such a way that the lid contained an opening with a diameter of 2.5 cm. This opening created a defined working area for the placement and processing of the implant. The bone blocks were inserted as far as they would go up until the sealing ring and the base was tightened with screws. A drainage system at the bottom contained two holes to prevent water accumulation and overflowing. The suction was then positioned at the border of the Plexiglas holder to the bone block.

### Artificial bone

An artificial 30 × 30 × 40 mm bone block made of polyurethane (open cell polyurethane foam PCF 20 pounds per cubic foot; Sawbones, Vashon Island) was used as a bone substitute. This was an enlarged model of a cancellous bone with open and closed pores.

### Protective materials

Three different materials were used as covering materials as off-label use. The protective cover was only applied to the top of the blocks around the implant up to over the edge to the holder.

#### Dental adhesive paste

The dental adhesive paste Solcoseryl (Meda Pharma GmbH, Vienna) is a drug to support the healing of damaged and inflamed oral mucosa. It contains a protein-free, standardized dialysate/ultrafiltrate derived from calf blood as the active ingredient and forms a protective film on the wound. Due to its very viscous and sticky consistency, it can be easily applied to the bone model with a Heidemann spatula and does not flow into the pores but forms a protective mass. For the samples of group 3, approximately 2.5 g of the paste was spread in each case so that it adhered tightly to the implant and the edge to the retainer.

#### Bone wax

Sterile bone wax (Bone wax; B Braun Surgical, Munich Germany) is used in surgery for mechanical hemostasis by closing intraosseous vascular channels. It consists of 70% beeswax and 30% Vaseline, and is non-absorbable. It retains a firm shape at room temperature but can be easily deformed and flattened by the heat of the hands. This allows it to be placed in one piece over the implant on the bone. Half a block (1.25 g) of bone wax was used for each of the samples in group 4.

#### Rubber dam with LC Block-Out Resin

Clinically, a rubber dam (Latex Rubber Dam, Ivory; Kulzer Mitsui Chemicals Group, Hanau, Germany) would normally be attached to the tooth with a rubber dam clamp. Since implants were used instead of teeth in this experiment and implantoplasty is often associated with horizontal and/or vertical bone defects, a more customized attachment method was needed. LC Block-Out Resin (Ultradent Products, Cologne, Germany), a liquid light-curing methacrylate-based resin, was used to secure and seal the rubber dam to the implant as apically as possible.

### Simulated treatments

The groups were divided into five groups which consisted of four test groups and one negative control group. The test group included three with protective covers and one positive control group. The test groups were treated with three protective materials, (1) rubber dam, (2) dental adhesive paste (DAP), and (3) bone wax, while four remained unprotected as a positive control. The fifth group (*n* = 3) was the negative controls.

#### Implantoplasty

The four test groups underwent a standardized implantoplasty procedure (Fig. 1b, 1I). The implant surface was machined for 2 min with a carbide drill (REF H379204014; Komet Dental, Lemgo, Germany) in a blue contra-angle handpiece at full speed (36,000 min^−1^) with water cooling. Afterwards, a diamond bur (REF 379,314,014; Komet Dental) was used in a red contra-angle handpiece (which) and the surface was finely polished again at full speed (180,000 min^−1^) with water cooling for 1 min. In the fifth negative control group, implantoplasty was skipped. All implants were treated by the same trained person (A.P.).

#### Preparation for measurements after implantoplasty

After removing the protective materials with a Heidemann spatula, the bone block was removed from the holder and placed in a Büchner funnel with filter paper (round filter 1288, 110 mm diameter, 0.21 mm thickness; Sartorius Faust Laborbedarf AG, Schaffhausen, Switzerland). The retention rate of the filter paper is 12–15 µm. The particle retention efficiency of a filter is expressed in terms of the particle size at which a retention content of 98% of the total number of particles is obtained. The cancellous bone was rinsed for 3 min with a jet of water from all sides to remove the penetrated titanium particles (TP) and collect them with the filter paper. The filter paper with TP in a 25-ml beaker was dried overnight in an oven at 37 °C.

After complete drying of the filter paper, the filter paper with TP was folded and put into a 25-ml beaker. Using a graduated pipette, 10 ml of 37% hydrochloric acid was measured, added to the beaker, and covered with a watch glass. Three samples fitted onto a magnetic stirring hotplate at a time. After dissolving and cooling the samples, they were put into a prepared 20-ml volumetric flask containing 200 µl of 10% calcium chloride and diluted to the 20-ml mark with Milli Q water. Since the resulting emulsion was still too viscous for the atomic absorption spectrometry instrument, the filter paper was separated from the sample. For this purpose, the emulsion was transferred from the volumetric flask to a centrifuge tube and centrifuged for 5 min at 4000 rpm (Heraeus Megafuge 8R; Thermo Fisher Scientific, Osterode am Harz, Germany). The clear supernatant was separated over the edge into a plastic cup and drawn up with a Luer Look syringe (Omnifix Luer Lock Solo, B Braun, Bad Arolsen, Germany) and filtered through a syringe filter (syringe filter, pore size 0.22 µm, 30 mm diameter; TPP Techno Plastic Products AG, Trasadingen, Switzerland) into a Greiner tube. The solutions were now ready for measurement.

### Titanium particle measurements

The titanium particles were measured by atomic absorption spectrometry (AAS ContrAA 300; Analytik Jena GmbH, Jena, Germany). The samples of groups 1 and 3 contained a higher concentration in the preliminary tests and therefore had to be diluted 10 times again (200-fold dilution in total). For this, 0.5 ml of the sample solution and 4.5 ml of a dilution solution (1 ml KCl 10%, 40 ml HCl 25% to 100 ml made up with Milli Q water) were mixed.

For the calibration of the instrument, a standard solution was prepared (Table 1). After measuring the standard solutions and calculating the regression, the remaining samples could be measured.

**Table 1:** Protocol for the preparation of a standard titanium solution for calibration and titanium quantity calculation (Cal Std = calibration standard)

	Titanium concentration (ppm)	Standard 1000 ppm Ti (µl)	KCl 10% (µl)	HCl 25% (ml)	Dilution with H_2_O (ml)
Cal Std 0	0.0	0	500	20	50
Cal Std 1	1.0	50	500	20	50
Cal Std 2	2.0	100	500	20	50
Cal Std 3	3.0	150	500	20	50
Cal Std 4	5.0	250	500	20	50
Cal Std 5	10.0	500	500	20	50
Cal Std 6	25.0	1250	500	20	50
Cal Std 7	50.0	2500	500	20	50

In addition to groups 1–5, a calibration sample with 19 mg TP was prepared in order to compare AAS with a precision balance. The titanium sample was dissolved using the same methods and served as a control for the titanium determination by AAS. From the 19 mg titanium, two samples could be prepared, both of which were measured.

### Additional investigations

#### Scanning electron microscopy

For a more detailed analysis, a bone sample with titanium particles and a control sample were examined under the scanning electron microscope (SEM). As preparation, the samples were glued to a SEM carrier with a carbon pad and sprayed with 10 nm gold. The SEM was operated at 10 kV.

In order to verify that they are indeed titanium chips, the chemical element titanium on the sample was determined by means of energy-dispersive X-ray spectroscopy (EDX) on the SEM. With the SEM, the size of the titanium particles can also be measured. A random sample was collected after implantoplasty.

#### Thickness of the protecting materials

The average layer thickness of the protecting materials was determined. For this purpose, the three materials were each applied to an acrylic block and measured with an electronic gauge (Digital Caliper, Toolland; Velleman Group, Belgium) and the mean value determined for each group (*n* = 3). The Sawbones bone model has a very irregular and porous surface which would lead to measurement inaccuracies. For this reason, a smooth acrylic block was used for these measurements. Furthermore, for each group (*n* = 1), the apically untreated implant area up to the acrylic block was measured after performing an implantoplasty. As a control group, an implant without covering materials was processed and measured.

### Statistical analysis

Excel (version 2202 Build 16.0.14931.20806) was used for coding and documenting the data. The data were analyzed with DATAtab Team (2022) (DATAtab: Online Statistics Calculator. DATAtab e.U. Graz, Austria. URL https:\\datatab.net). The normality of the data distribution was tested with the Kolmogorov–Smirnov and Shapiro–Wilk test. Descriptive statistics were used to describe mean, median, standard deviation, and IQR. The non-parametric Kruskal–Wallis and Mann–Whitney tests were used to determine significant differences between the groups studied. For all statistical tests, a significance level *p* < 0.05 was defined as statistically significant.

## Results

### Titanium quantity after implantoplasty

**Fig. 2:**
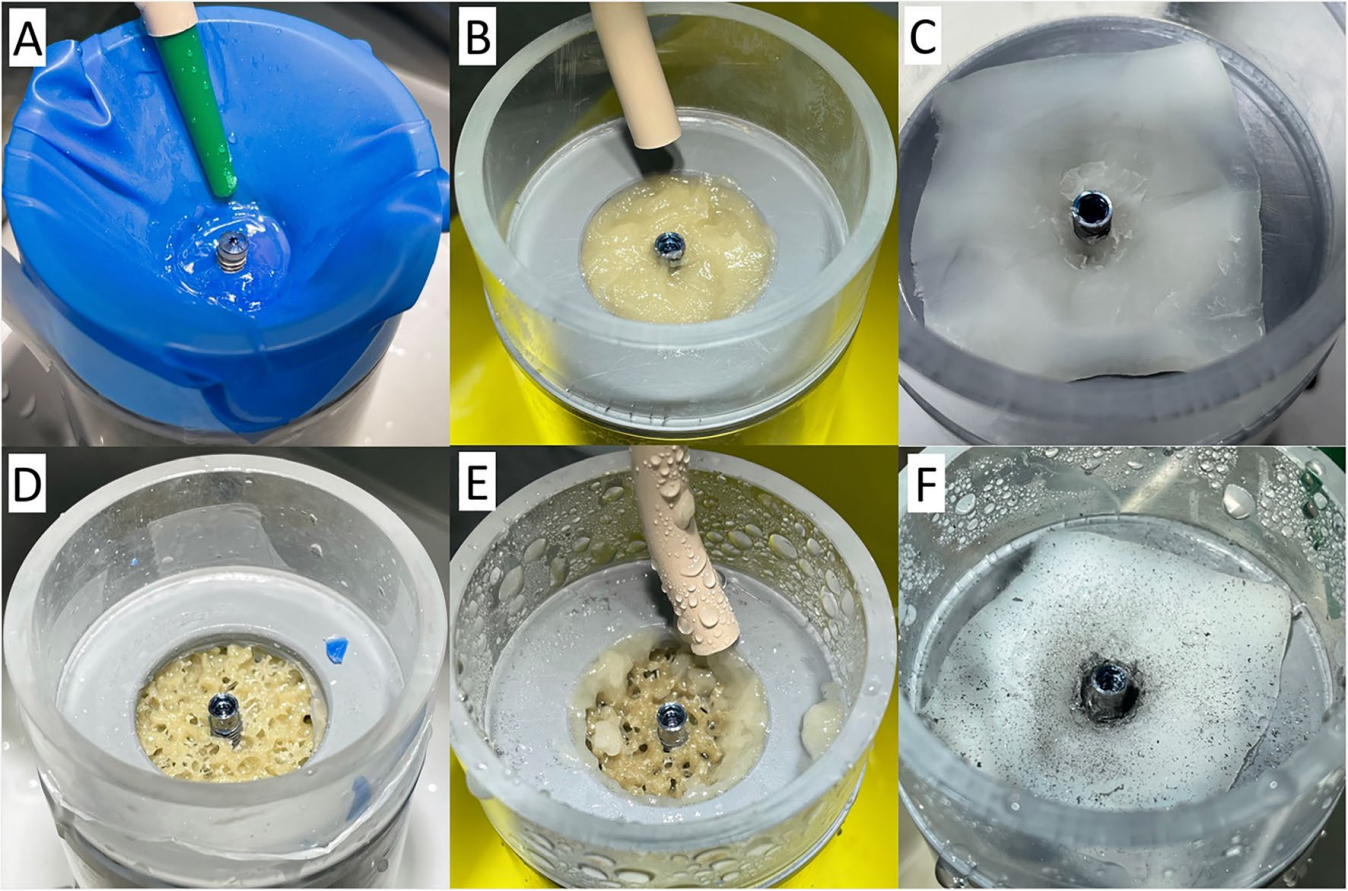
Experimental set-up with protective materials before (**A**–**C**) and after implantoplasty (**D**–**F**). After implantoplasty, the underlying bone under rubber dam protection showed no visible particles (**D**). In contrast, the adhesive paste was diluted and removed during implantoplasty, making the bone accessible to the titanium particles, which are visible in (**E**). Bone wax was more stable during wet treatment; titanium particles were visible on the barrier material, which visually seemed to adequately protect (F)

**Fig. 3:**
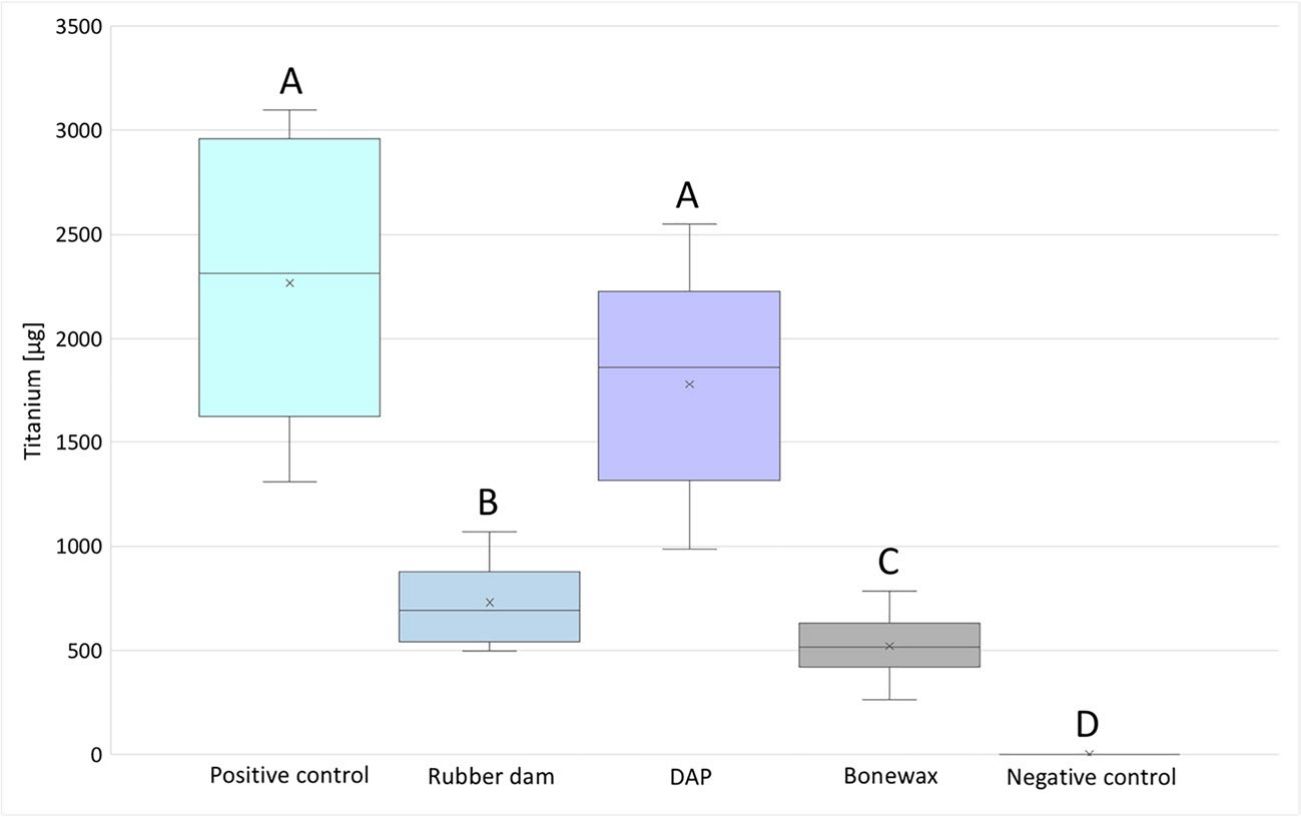
Measured titanium amounts as box-plot illustration after implantoplasty. The values that differed significantly from each other are marked with different capital letters

The amount of titanium after implantoplasty differed significantly in the rubber dam (*p* < 0.001), bone wax (*p* < 0.001), and negative control (*p* < 0.001) groups from the positive control (Table 2). The dental adhesive paste (DAP) group did not differ significantly from the positive control (*p* = 0.19) (Fig. 3). The most amount of titanium was measured in the positive control with no protective measure (2313 ± 746.87 µg), followed by DAP (1863.5 ± 537.51 µg). The rubber dam (691 ± 248.64 µg) and bone wax (516 ± 156.58 µg) groups had significantly lower values. Thus, the rubber dam and bone wax groups showed to have the best shielding effect of the bone from titanium particles. Figure 2 shows the three different types of protective materials before and after implantoplasty.

**Table 2:** Summary of the descriptive results after the different treatments and controls (µg)

	Control +	Rubber dam	DAP	Bone wax	Control −
Mean value	2265.9	730	1778.4	520.8	0
SD	746.87	248.64	537.51	156.58	0
Median	2313	691	1863.5	516	0
Quartile 1	1662.75	564	1440.5	451.25	0
Quartile 3	2959	810.5	2139.5	590.5	0
IQR	1336.25	246.5	699	139.5	0
95% CI	1802.84; 2728.96	601.09; 2111.55	1445.25; 2111.55	423.75; 617.85	0; 0

The two samples used as calibration with 19 mg of titanium had a titanium content of 11.09 mg and 11.011 mg titanium in the AAS measurement (Table 3).

**Table 3:** Determination of titanium with atomic absorption spectrometry (AAS) 19 mg titanium measured by precision balance which served as a control of the measurement procedure

Sample	Ext	Ti ppm (µg/ml)	Dilution	Ti/sample (µg)
19 mg Ti 100 × 1	0.00238	5.545	2000	11,090
19 mg Ti 100 × 2	0.00219	5.055	2000	10,110

### Titanium samples with SEM/EDX

**Fig. 4:**
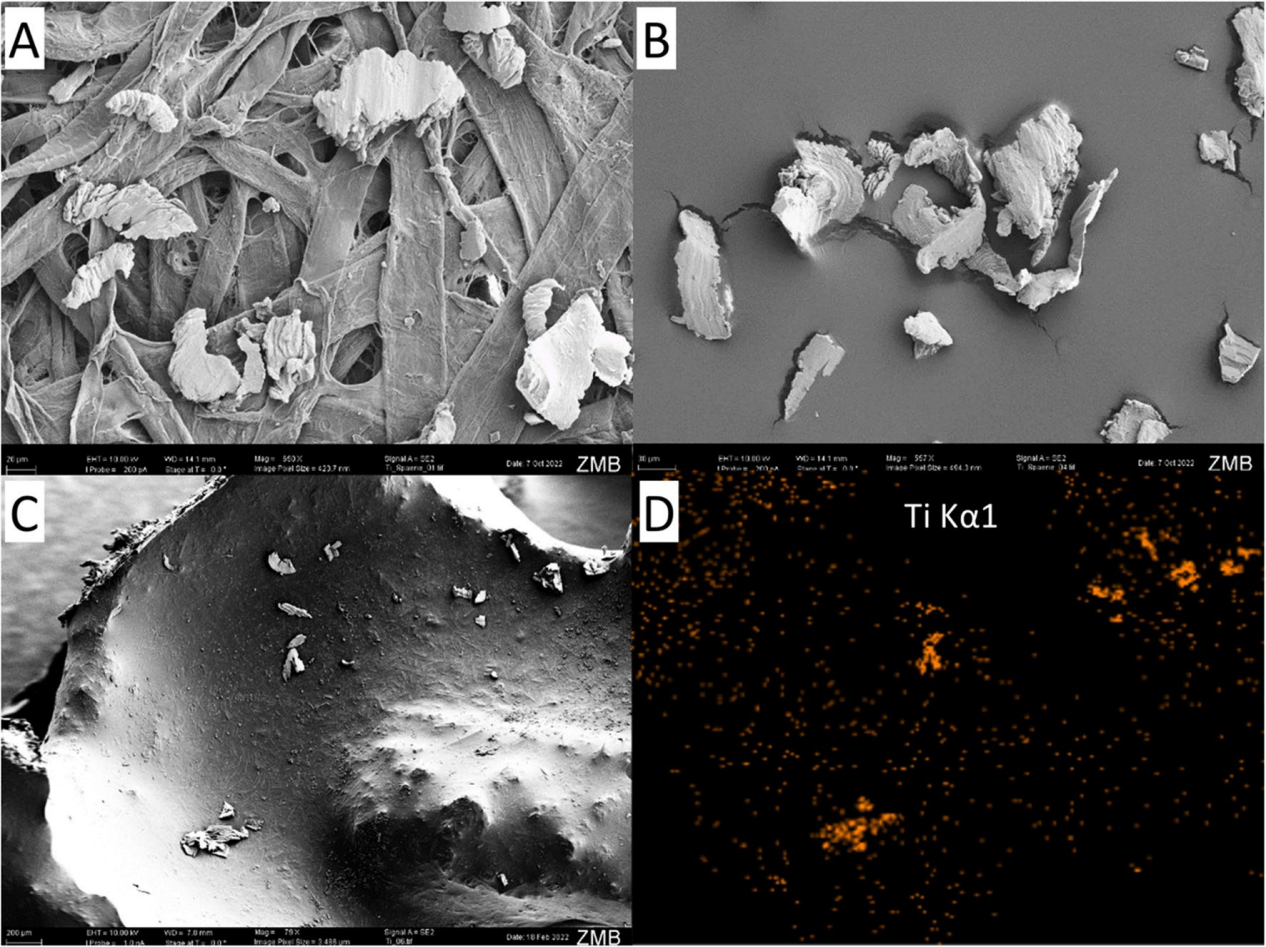
Titanium particles on a filter paper (**A**) and on a carbon pad (**B**) under scanning electron microscopy (SEM). Also, in the artificial bone model (**C**), particles were visible, which were identified as titanium by energy-dispersive X-ray spectroscopy (**D**)

On the bone model with titanium, individual titanium particles can be seen, which were also identified as titanium by the EDX. The control sample had a rather rough surface with small impurities (Fig. 3). Since the bone model has a very prominent three-dimensional structure with curvatures and holes, the EDX detector could not detect all signals due to shadows. On flat surfaces, however, the particles could be identified as titanium as is shown in Fig. 4. Regarding the size of the particles, it was found that particles < 10 µm were also produced during implantoplasty. It can therefore be assumed that not 100% of the titanium particles produced could be collected by the filter paper used.

### Thickness of the protective materials and untreated surface width

The average layer thickness was 1.97 mm for bone wax, 2.41 mm for dental adhesive paste, and 2.4 mm for rubber dam. In the implantoplasty, the apical 0.15 mm was not processed in the positive control (without covering), which can be attributed to the shape of the instruments/burs used. In the bone wax 1.19 mm and in the rubber dam 0.78 mm of the implant were not processed. Since the dental adhesive paste gets blown away quickly, only 0.26 mm of the apical part of the implant was not treated. With bone wax and rubber dam, on the other hand, accessibility is impaired by approximately 1 mm due to the protective material.

## Discussion

In the present in vitro study, the protective potential of three different types of barrier materials to shield the peri-implant bone from titanium particles during implantoplasty were evaluated. These techniques may serve as a measure to reduce and ensure no new debris or particles would enter the peri-implant tissues caused by the treatment and thus might prevent the triggering of potential inflammatory reactions.

None of the three protective materials tested could completely prevent titanium particles from penetrating the bone model during implantoplasty. Rubber dam and bone wax showed a significant reduction of titanium particles. Dental adhesive paste did not show a significant reduction. From the study, it does not seem to be suitable for shielding the bone because it is immediately washed away by the water–air flow of the contra-angle handpiece and squeezed into the bone pores. It is therefore suspected that the titanium particles could stick to the bone through the paste, as they can no longer be removed from the pores. With the bone wax, the titanium particles were also pressed into the material, but the barrier was not broken. The bone wax can be easily removed from the model when cooled. Whether water cooling is also sufficient in a warm oral cavity would still have to be evaluated clinically. Bone wax has been associated with foreign body reactions in various surgical specialties [17]. Therefore, if used, removal of the material without residue should be ensured.

Surgical peri-implantitis treatment with implantoplasty which involves the mechanical removal of implant threads to have a smooth surface to reduce eventual biofilm regrowth and accumulation have been performed with good clinical outcome especially in reduction of BoP over the years [18–20]. However, this process results in a significant release of debris to the surrounding tissues.

The in vitro inflammatory response of peri-implant granulation tissue fibroblasts to titanium particles has been described alone and in the presence of *Porphyromonas gingivalis* (*Pg*). A study reported that titanium particles in the peri-implant tissue in combination of a *Pg* infection contribute to the pathogenesis of peri-implantitis by increasing the inflammatory response [21]. Studies have shown that titanium and metal debris in the soft tissues can induce pro-inflammatory responses in the tissues, such as expression of inflammatory cytokines, and decrease viability of osteogenic cells [22, 23]; activation of osteoclasts and morphologic alterations might occur in cells such as neutrophils and macrophages [8, 14, 24]. An in vitro study demonstrated reduced viability of gingival fibroblasts cultured in the presence of implantoplasty debris [25]. Titanium particles have also been shown to trigger DNA damage response pathway and disrupt epithelial homeostasis in oral epithelial cells [26]. Furthermore, metal particles released by implantoplasty resulted in pro-inflammatory effects and decreased expression of osteogenic markers [27].

Although the treatment of peri-implantitis with implantoplasty shows significant improvements [6], there are also critical voices who do not recommend implantoplasty based on their studies of micro- and nanoparticle release from dental implants. The full systemic effect has not been fully clarified [25].

In addition, it is also important to consider the effect of the size and surface area of the released particles which has been demonstrated to influence negatively on the biological behavior of the cells [22, 28–31].

Therefore, the need for careful evaluation of peri-implantitis treatment is of importance due to the release and risks of potential cytotoxicity of titanium alloy particles. Calls for further studies to improve methods to prevent and reduce the number of titanium particles into the surrounding tissues are needed.

The limitation of this study is that the bone model that was used had a much larger pore size (PCF 20) than in the human cancellous bone and thus is not a true replication of the human biological condition. Moreover, not all materials could be removed without any residue. The question arises whether these material residues themselves could also cause inflammatory reactions. Due to the shape of the instruments and the protective materials, the most apical part of the exposed implant could not be treated and thus retains the rough surface where bacteria can accumulate. The extent to which this approximately 1 mm of the untreated implant has an influence on the clinical outcome must be investigated in further trials.

## Conclusion

In conclusion, taking into account the discussed limitations of the model, it can be assumed that the titanium particles generated by implantoplasty can be significantly reduced by the protective materials rubber dam and bone wax. In view of the many indications of the pro-inflammatory effect of titanium particles in bone and soft tissue, these initial results speak in favor of further research into possible protective materials and methods.
